# Neurocognitive effects of 3 mA prefrontal electrical stimulation in schizophrenia: A randomized sham-controlled tDCS-fMRI study protocol

**DOI:** 10.1371/journal.pone.0306422

**Published:** 2024-08-16

**Authors:** Amir-Homayun Hallajian, Kiomars Sharifi, Reza Rostami, Fahimeh Saeed, Shirin Mokarian Rajabi, Negin Zangenehnia, Zahra Amini, Zahra Askari, Fidel Vila-Rodriguez, Mohammad Ali Salehinejad

**Affiliations:** 1 Department of Psychology, University of Tehran, Tehran, Iran; 2 School of Cognitive Sciences, Institute for Research in Fundamental Sciences (IPM), Tehran, Iran; 3 Psychosis Research Center, University of Social Welfare and Rehabilitation Sciences, Tehran, Iran; 4 School of Biomedical Engineering, University of British Columbia, Vancouver, Canada; 5 Department of Psychiatry, University of British Columbia, Vancouver, Canada; 6 Department of Psychology and Neurosciences, Leibniz-Institut fur Arbeitsforschung, Dortmund, Germany; Gabriele d’Annunzio University of Chieti and Pescara: Universita degli Studi Gabriele d’Annunzio Chieti Pescara, ITALY

## Abstract

**Background:**

Schizophrenia (SCZ) is characterized by cognitive deficits that are linked to prefrontal cortex dysfunction. While transcranial direct current stimulation (tDCS) shows promise for improving cognition, the effects of intensified 3mA tDCS protocols on brain physiology are unknown. This project aims to elucidate the neurophysiological and cognitive effects of an intensified prefrontal tDCS protocol in SCZ.

**Methods:**

The study is designed as a randomized, double-blind, 2-arm parallel-group, sham-controlled, trial. Forty-eight participants with SCZ and cognitive impairment (measured via a set of executive functions tests) will be randomly allocated to receive either a single session of active (n = 24) or sham (n = 24) tDCS (20-min, 3-mA). The anodal and cathodal electrodes are positioned over the left and right DLPFC respectively. The stimulation occurs concurrently with the working memory task, which is initiated precisely 5 minutes after the onset of tDCS. Structural and resting-state (rs-fMRI) scans are conducted immediately before and after both active and sham tDCS using a 3 Tesla scanner (Siemens Prisma model) equipped with a 64-channel head coil. The primary outcome will be changes in brain activation (measures vis BOLD response) and working memory performance (accuracy, reaction time).

**Discussion:**

The results of this study are helpful in optimizing tDCS protocols in SCZ and inform us of neurocognitive mechanisms underlying 3 mA stimulation. This study will additionally provide initial safety and efficacy data on a 3 mA tDCS protocol to support larger clinical trials. Positive results could lead to rapid and broader testing of a promising tool for debilitating symptoms that affect the majority of patients with SCZ. The results will be made available through publications in peer-reviewed journals and presentations at national and international conferences.

## 1. Introduction

SCZ (SCZ) is one of the most severe neuropsychiatric disorders, with poor response or adherence to current treatment strategies in 40% of patients [1]. In the latest Global Burden of Disease Study, SCZ is among the top 20 diseases in 2021 in disability metrics [2]. Cognitive impairments, particularly in working memory and executive functions, are more pronounced than positive symptoms like hallucinations and delusions in SCZ [3–5]. These impairments are associated with significant functional impairment and poor prognosis. SCZ is characterized by frontal abnormalities, especially in the prefrontal cortex (PFC), which are linked to executive dysfunction and working memory deficits [5–7]. Consequently, interventions targeting cognitive control through PFC activity modulation have been suggested as a possible treatment for SCZ [4, 8] and other disorders with frontal problems [9–13]. Transcranial direct current stimulation (tDCS) is a safe and noninvasive brain stimulation technique for modulating brain excitability [14, 15] and has been increasingly applied in SCZ for cognitive benefits [8, 16]. Despite promising preliminary results in SCZ, the mechanisms underlying the observed effects and systematic evaluation of intervention protocols that can lead to clinically optimal effects have not been well explored.

While non-invasive brain stimulation techniques are safe methods for directly modifying brain functions in humans and studying the causality of brain-behavior relationships [17], neuroimaging methods, including functional magnetic resonance imaging (fMRI), enable the study of brain function in vivo. Combining these methods allows for experimental induction of alterations in neural processes that have impacts on cognition and underlying physiological processes, as well as assessing cognitive brain correlations [17, 18]. the cognitive benefits of tDCS in SCZ partly rely on the dosage of stimulation [4, 19], which is also observed in healthy populations [20]. Recent physiological findings suggest that an intensified protocol with anodal tDCS (3 mA) can induce longer-lasting and stronger aftereffects on motor cortex plasticity [21], which is also transferred to the prefrontal cortex [22]. Furthermore, for the cognitive effects of stimulation, it is important to consider its effects on functional networks [23]. While previous studies have shown that tDCS can improve cognition in a dose-dependent manner in SCZ [19], the physiological mechanisms underlying these effects have not been fully elucidated. Given the importance of stimulation intensity for cognitive outcomes, it is relevant to evaluate the efficacy of an intensified 3mA tDCS protocol, which has not yet been studied in SCZ, to the best of our knowledge.

This study was designed to investigate the effects of online 3-mA prefrontal tDCS on working memory and the associated brain physiology in patients with SCZ. Specifically, the objectives of the present study were to examine (1) changes in working memory task performance during online 3-mA tDCS in SCZ, (2) changes in blood-oxygen-level-dependent (BOLD) response in the targeted regions after 3-mA tDCS, (3) changes in functional connectivity of executive control networks after 3-mA tDCS, and (4) associations between changes in performance and functional connectivity in patients with SCZ. We hypothesized that compared to sham, 3-mA prefrontal tDCS will improve working memory performance, increase frontal activation, and strengthen executive network connectivity. We also expect that those with greater improvements in connectivity will exhibit better performance. Investigating the neurophysiological effects of intensified tDCS can inform the optimization of tDCS protocols for SCZ.

## 2. Methods and analysis

### 2.1 Trial design

**Fig 1:**
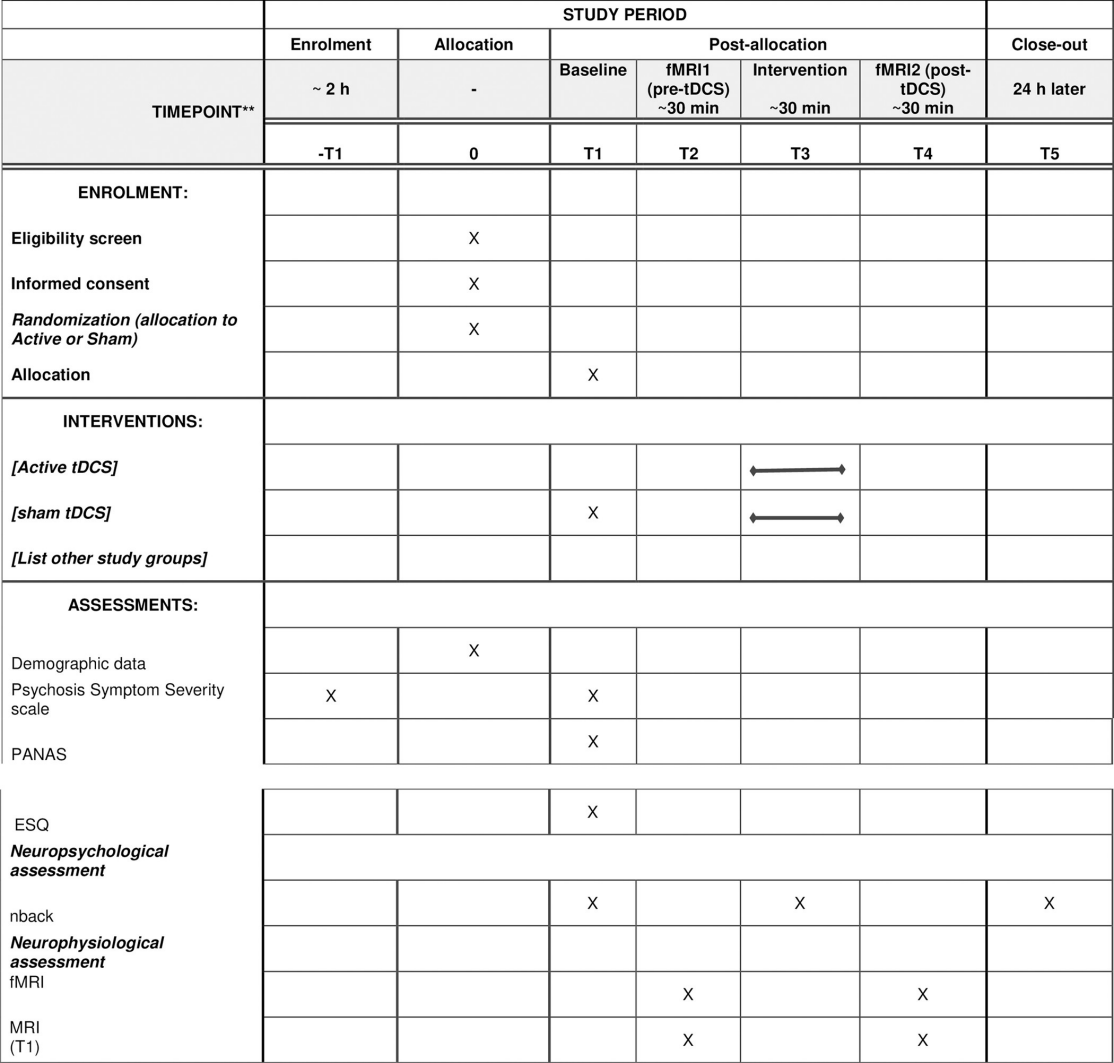
SPIRIT schedule. Note: PANAS: Positive Scale, Negative Scale, and General Psychopathology Scale; ESQ: Executive Skills Questionnaire for Adults.

The study design and schedule were reported in accordance with the SPIRIT guidelines [24] (Fig 1). In this study, we conducted a randomized, double-blind, parallel-group, sham-controlled investigation at the National Brain Mapping Laboratory and Institute for Research in Fundamental Sciences in Tehran, Iran, in collaboration with the Department of Psychiatry at the University of British Columbia in Canada and the Department of Psychology and Neurosciences at ifado, Germany. Our report adhered to the Consolidated Standards of Reporting Trials (CONSORT) statement for non-pharmacological treatments [25]. Currently, we are in the recruitment phase, aiming to enroll a cohort of 48 participants diagnosed with SCZ Spectrum and Other Psychotic Disorders, according to the DSM-5 criteria, by a licensed psychiatrist. These participants will fall within the age range of 18–50 years. To ensure the scientific rigor of our research, we will systematically and randomly allocate these participants into one of two parallel groups. Each group will receive either active or sham tDCS. Importantly, half of the participants will undergo a single tDCS session, which involves anodal and cathodal stimulation over the left and right Dorsolateral Prefrontal Cortex (DLPFC) respectively. The other half will receive sham stimulation as part of this research study.

**Fig 2:**
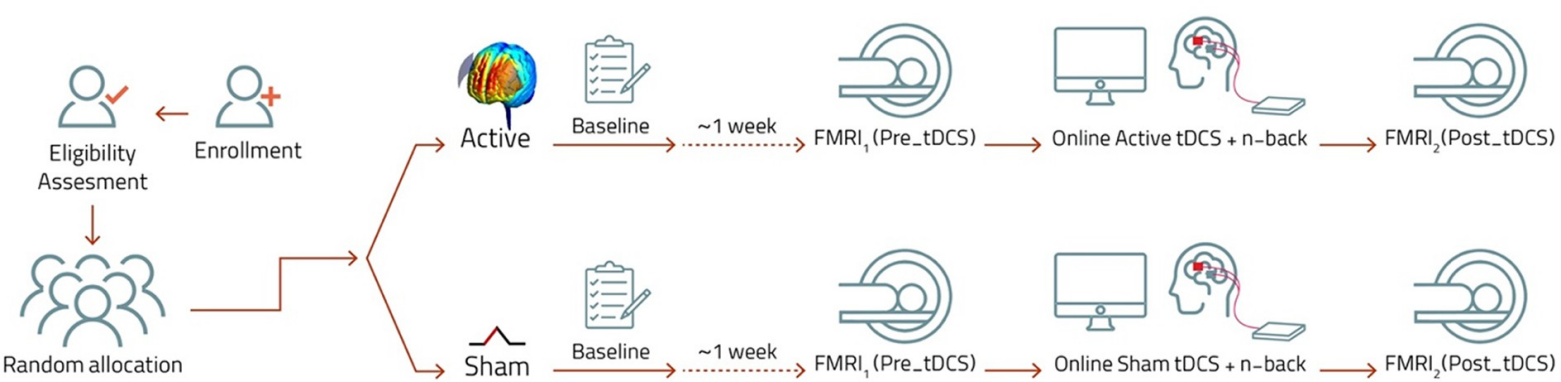
Course of study overview.

Eligible participants, after confirming inclusion criteria and obtaining informed consent, undergo comprehensive baseline assessments. These assessments include reconfirming the diagnosis using the Clinician-Rated Dimensions of Psychosis Symptom Severity [26], administered by a trained psychiatrist. Additionally, participants complete the native language versions of the Montreal Cognitive Assessment (MoCA-P) scale [27] and Executive Skills Questionnaire for Adults (ESQ) [28, 29], and their symptomatology is evaluated using the Positive Scale, Negative Scale, (PANSS) [30, 31]. The cognitive screening and clinical assessment will be conducted with native language versions of each scale with sufficient psychometric features. At least one week prior to participating in the intervention session (tDCS/fMRI), a baseline assessment of working memory is conducted using the n-back test. During the intervention session, participants are first checked for sleep pressure as it greatly impacts cortical excitability, tDCS-induced plasticity and cognitive performance [32]. Then, participants in the active group undergo online anodal tDCS at 3 mA for 20 minutes. The anodal electrode is positioned over the left DLPFC while the cathode is placed over the right DLPFC. This stimulation occurs concurrently with the working memory task, which is initiated precisely 5 minutes after the onset of tDCS. Brain stimulation is conducted in a room next to the scanner and structural and resting-state (rs-fMRI) scans are conducted immediately before and after both active and sham tDCS (Fig 2). To keep the time interval stable, we start the post-stimulation rs-fMRI 5 minutes after the end of stimulation to move the subject on the scanner bad and prepare it.

### 2.2 Ethics committee and regulatory approval

The trial will be conducted in accordance with the ethical principles outlined in the Declaration of Helsinki 2013. This research has received approval from the Research Ethics Committees of Iran University of Medical Sciences under Approval ID: IR.IUMS.REC.1401.02 and prospectively registered with clinicaltrials.gov under the identifier NCT05200962. In the event of any severe side effects occurring during the trial, they will be promptly reported to the safety monitoring board and the Institutional Review Board (IRB) for appropriate management.

### 2.3 Participants and recruitment

We aim to recruit 48 participants from specialized clinics affiliated with Razi Psychiatric Hospital in Shahre-Rey, Iran, using flyer advertising and referrals from their treating psychiatrists. The exclusion of smokers is not feasible because a significant proportion of patients with SCZ are smokers. The expected population is SCZ patients with cognitive impairment (see inclusion criteria) who are not in a severe stage. Below are the inclusion and exclusion criteria:

Inclusion Criteria:

To be eligible for participation, individuals must meet the following criteria

1. Age: Men or women aged 18–55 years.
2. Diagnosis of schizophrenia or schizoaffective disorder according to DSM-5 with the native language version of the DSM-5 Clinician-Rated Dimensions of Psychosis Symptom Severity [33] and the native language version of the Positive and Negative Syndrome Scale [31] conducted by two skilled licensed psychiatrists. Patients with disorganized or negative motor behaviors will not be included.
3. Cognitive Impairment: Should exhibit significant cognitive impairment, defined as performance of at least one standard deviation below the norm on working memory/executive functioning tasks measured by a native language version [27] of the Montreal Cognitive Assessment [34].
4. Feasibility: Must be suitable for tDCS interventions following safety guidelines.
5. Treatment with stable doses of antipsychotics and all CNS-activating medications (4–6 weeks before the experiment). For the purpose of this study, stability is defined as a change of not more than 50% in the antipsychotic dose.
6. Right-Handedness
7. Informed Consent: Written and witnessed informed consent (the informed consent is provided by independent research assistants and not the treating psychiatrist to avoid coercive treatment)
8. Participants must read and write in Persian at a level sufficient to understand and complete study-related procedures

Exclusion Criteria:

Participants will be excluded from the study if they meet any of the following criteria.

1. Pregnancy
2. Substance Dependence
3. Presence of other neuropsychiatric disorders except for schizoaffective disorders
4. Seizure History
5. Neurological Disorder
6. Head Injury
7. The presence of ferromagnetic objects in the body that are contraindicated for MRI of the head (pacemakers or other implanted electrical devices, brain stimulators, some types of dental implants, aneurysm clips, metallic prostheses, permanent eyeliners, implanted delivery pumps, or shrapnel fragments), or fear of enclosed spaces).

### 2.4 Plans to promote participant retention

To enhance participant engagement and alleviate anxiety, a video explaining the project’s execution process, including details about the MRI environment and sounds, is presented by a qualified psychiatrist one week before the intervention. This video includes a mock model of a 3 Tesla scanner (Siemens Prisma) to familiarize participants with the MRI atmosphere. They are encouraged to ask questions and express concerns. On the testing day, a team of trained researchers welcome the participants and they remained with them throughout the experiment. Any adverse effects are diligently recorded, and participants are provided with contact information of the research team psychiatrist in case of post-study side effects. Participants are informed of their right to withdraw from the study at any time, with the assurance that their data will be excluded from the final results if they choose to do so.

### 2.5 Sample size estimation

The sample size is determined a priori through power analyses via G*Power 3.1 [35], indicating that a minimum of 42 subjects is required to achieve 95% power at a significance level of 0.05 for the primary statistical test of a mixed-model ANOVA design, with two groups, 3 measurements of working memory performance (baseline, during stimulation and 24 hours after stimulation), given a medium effect size (f = 0.25, equivalent to η^p2^ = 0.10 which is used in ANOVA models). This effect size is recommended in NIBS studies [36]. To account for a potential dropout rate during the study, we propose including an additional 3 participants in each group (i.e., 6), totaling 48, which is more than the majority of published studies in terms of sample size and retention rate [37, 38].

### 2.6 Blinding

During the participant recruitment phase, potential participants are informed that the study is focused on evaluating neural responses to tDCS. However, no specific information has been provided about the existence of sham tDCS or the distinctions between these two types of stimulation. To preserve the integrity of blinding, the tDCS device’s screen remains concealed from participants, and the allocation of participants into the experimental or sham group is undisclosed to both the participants and the researchers. Furthermore, the application of tDCS is carried out by a technician who is unaware of the research objectives. In the sham tDCS condition, there is a 30-second ramp-up to 3 mA, immediately followed by a 30-second ramp-down to zero intensity. This design is intended to create a sensation similar to that of a real stimulation without actual delivery. To assess the effectiveness of blinding, participants will be queried at the study’s conclusion to make an informed guess about whether they received real or sham stimulation.

### 2.7 Randomization

To ensure unbiased participant allocation, we will use an online block randomization tool (https://www.sealedenvelope.com/simple-randomiser/v1/lists) with a block size of six, dividing participants into two treatment groups. The allocation process will be concealed using unique codes, which will be sealed within opaque envelopes. These envelopes will be prepared in advance by a research assistant who is not directly involved in this study. The intervention will be administered by separate investigators who are not linked to the assessment of the outcome measures. Additionally, the experimenter responsible for conducting the outcome measures will remain blinded to the tDCS conditions.

### 2.8 Intervention

**Fig 3:**
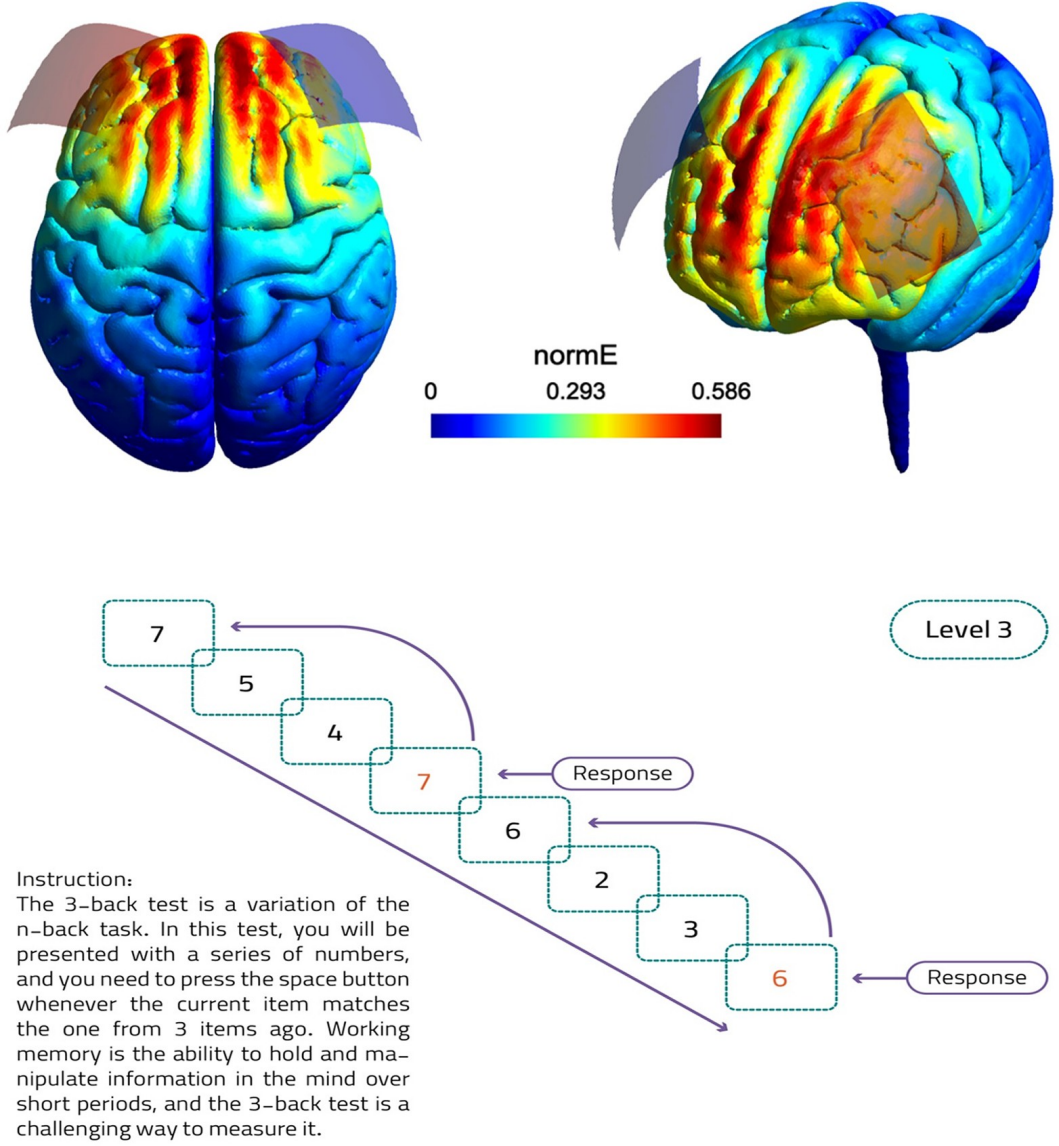
a) Modeling of induced Electrical field of 3 mA bilateral frontal montage with anode in left DLPFC and cathode in right DLPFC (red rectangle: anode, blue rectangle: cathode), b) Illustration of the 3-back test and instructions to the participants as an example.

tDCS will be administered using the DC-Stimulator Plus device (Neuroconn GmbH, Germany). A constant current of 3 mA will be applied for a duration of 20 minutes, with 30-second ramp-up and ramp-down periods, delivered between two sponge electrodes, each measuring 7 x 5 cm^2^, soaked in saline solution. The anode electrode will be positioned over the left DLPFC (F3 based on the 10–20 EEG system) and the cathode electrode will be situated over the DLPFC (F4). This bilateral electrode arrangement is chosen to primarily increase excitability over the left DLPFC in line with previous tDCS studies in the SCZ [38, 39]. The placement of the return electrode (here cathodal) has been different (e.g., right DLPFC, orbitofrontal cortex, temporoparietal junction) in previous tDCS studies but was mostly on the right hemisphere [38]. We place the cathode over the right DLPFC as the DLPFC is the primary target here and of interest for BOLD response following tDCS. In the sham control condition, the same electrode positions and ramping periods will be employed, but a 3-mA current will only be delivered during the initial minute of the 20-minute session to induce a temporary tingling sensation on the scalp, ensuring participant blinding. Participants will be informed of the possibility of experiencing mild tingling or itching sensations resulting from the electrodes’ contact with the scalp. To prevent skin irritation due to the 3-mA current and also prevent unblinding, an anesthetic cream will be applied under the electrodes and over the scalp. Following each active or sham session, participants will complete a checklist to record any adverse effects. The electrical field induced by this protocol is modeled in Fig 3 using the SimNIBS software [40]. The stimulation will be delivered online, that is, during cognitive task performance. After the stimulation and at the end of the experiment, a side effect survey on tDCS common side effects (e.g., itching, tingling, burning, pain sensations) will be conducted. In the event of a significant increase in the risk of serious adverse events, such as severe itching or redness at the electrode sites, or if safety concerns arise from the Research Ethics Committee of Iran University of Medical Sciences, the investigators will have the authority to halt the trial.

### 2.9 Magnetic resonance imaging

MRI assessments will be performed using a Siemens MAGNETOM Prisma 3 Tesla scanner, equipped with a 64-channel head coil. These assessments will take place both before and immediately after the intervention. Each MRI session will encompass whole-brain structural images with T1-weighted acquisition and rs-fMRI scans. Each MRI session will include the acquisition of whole-brain structural images and resting-state fMRI (rs-fMRI) scans. The T1-weighted images will be acquired over five minutes using MPRAGE pulse sequences with parameters set at a TR of 1800 ms, TE of 3.53 ms, a voxel size of 1 × 1 × 1 mm^3^, and a flip angle of 7°. Additionally, rs-fMRI will be conducted for 15 minutes using an EPI sequence with a TR of 2000 ms, TE of 30 ms, a voxel size of 3 × 3 × 3 mm^3^, and a flip angle of 90°.

### 2.10 Outcome measures and assessments

#### 2.10.1 Working memory task

To assess working memory performance, we will employ the N-back task, a widely recognized ’executive’ assessment for working memory (WM) (Fig 3) related to the prefrontal cortex (PFC) [41]. This task requires continuous monitoring and updating of the remembered information. Participants will be presented with a sequence of numbers one at a time and are instructed to indicate when the current number matches the one presented n” steps earlier in the sequence. The working memory demand increases with higher ’n-back’ levels. In our study, participants will engage in both 1-back and 3-back conditions in line with previous works [42], once prior to the intervention (tDCS/fMRI) session for baseline assessment and once during the online stimulation. In the 1-back task, participants identify when the current number stimulus matched the one immediately preceding it, placing minimal demand on the working memory. In the more challenging 3-back, participants must recognize when the current stimulus matches the one shown three steps back, necessitating the maintenance and manipulation of information in working memory.

The N-back test will be implemented using MATLAB (MathWorks, Inc., 2019) and the PsychToolbox library. The experiment comprised 120 trials, with each stimulus (a digit between 0–9) displayed for 0.5 seconds followed by an inter-trial interval (ITI) that was dependent on the N-back level, calculated as 2 + 0.25 * (N_back_-1) seconds. Participants were prompted to respond by pressing the ’Space’ key for N-back instances. During the training stage, a shorter version with visual feedback will be applied, where the screen turns red when the wrong button is pressed, and green for the correct response. Outcome measures include accuracy, reaction time for correct responses (ms), and d-prime—a bias-free measure of observer sensitivity [43] considering both correct hits and false alarms. D-prime is calculated as

d′=z(Hitrate)−z(Falsealarmrate)

Although our study design did not initially include direct measures of response bias through differentiated response buttons, we recognize its importance. We propose evaluating the response bias using the criterion C = −0.5[z(hit rate)+z(false alarm rate)], as suggested by Huh et al. (2006) [44], to infer participants’ response tendencies from our data, despite the constraints of our data collection protocol. A comparison of 1-back and 3-back performances will provide a robust characterization of working memory abilities. Before the start of the baseline block, participants will perform continuous training on both 1 and 3-back tasks until they reach a stable level of performance (>70% accuracy).

#### 2.10.2 Primary outcomes

The primary outcome measures are:

1. change in the d’ index of the n-back task from baseline to the online stimulation session, which will be assessed in both the sham and active tDCS groups and 24 hr after the intervention
2. Change in the accuracy of the n-back task from baseline to the online stimulation session and 24 hr after the intervention
3. Change in the reaction time of correct responses of the n-back task from baseline to the online stimulation session and 24 hr after the intervention
4. Changes in rs-fMRI functional connectivity by measuring correlations in spontaneous BOLD signal fluctuations between cortical and subcortical regions of interest (ROIs) before and after tDCS administration
5. Changes in rs-fMRI functional connectivity by examining correlations between spontaneous BOLD signal fluctuations in the stimulated cortical area under the anode/cathode electrodes and the entire brain, both before and after tDCS.

#### 2.10.3 Exploratory outcomes

We will perform exploratory analyses to investigate the correlations between changes in physiology and changes in cognitive performance before and after tDCS. This approach allows us to assess the intricate relationship between brain function and behavior. Furthermore, our objective is to identify potential predictors of variations in working memory task performance and individual responses to the intervention, taking into account a range of clinical and biological measures. In addition, we will incorporate the induced electric field (E-field) in the prefrontal area for each participant. This E-field is determined through an analysis of the computational finite-element model with simNIBS software and is considered as a potential predictor of how the brain responds to tDCS.

### 2.11 Data management

The research team consists of the principal investigator, a trained psychiatrist, professional researchers, and research assistants. Monthly meetings are held to maintain project administration consistency, address critical matters, and monitor trial progress. The psychiatrist directly communicates with the patients. Two professionally trained researchers are tasked with collecting and inputting verified data, all while being supervised by MA Salehinejad.

### 2.12 Statistical methods

To assess the efficacy of the tDCS intervention, we will conduct a mixed model analysis of variance (mixed-ANOVA) on the n-back task indices, including d-prime, reaction time, and accuracy with time (before and after tDCS) as the within-subject factor and group (active tDCS and sham tDCS) as the between-group factor and their interaction. The group × time interaction effect and the main effect of group and time will be explored to see if changes in working memory performance and brain activation (two key dependent variables) are influenced by the type of stimulation (group factor) and time (before vs during stimulation). The normality and homogeneity of data distribution, and variance will be confirmed by Shapiro-Wilk and Levin tests, respectively. Mauchly’s test will also be used to evaluate the sphericity of the data before performing the ANOVA. In case the assumption of sphericity was violated, the degrees of freedom were corrected using Greenhouse-Geisser estimates of sphericity. To investigate the associations between changes in brain activity/connectivity and task performance changes, we will employ the Pearson correlation test. This test will help identify potential relationships between changes in brain activity and task performance, providing valuable insights into the neural mechanisms underlying our observations. All statistical analyses will be conducted using R [45].

We will preprocess rs-fMRI data using the fMRIPrep Pipeline [46] and analyze functional connectivity with the FSL software [47]. Our preprocessing steps encompass standard procedures, including slice-timing correction for synchronization across slices, realignment for motion correction, unwarping, spatial normalization, and smoothing via Gaussian kernel application. Additionally, we employ Nuisance Regression, which involves regressing out signals originating from non-neural sources such as white matter, cerebrospinal fluid (CSF), and global signal [48–50]. These strategies aim to mitigate confounding factors and enhance the reliability of our findings. Our analysis specifically targets the DLPFC due to its direct stimulation by tDCS, and will extend to the Default Mode, Salience, and Executive Control Networks, which are crucial for understanding schizophrenia’s cognitive aspects. We will employ seed-based correlations and ICA to identify coherent functional networks, ensuring a comprehensive exploration of brain connectivity. Multiple imputation will be the primary analysis strategy for addressing missing data (with the presence of more than 5% missing data) by employing fully conditional specification and predictive mean matching to generate ten imputed datasets. These imputed datasets will be used for the main analysis. The analysis will be conducted on each imputed dataset, and results will be combined following Rubin’s rules to provide comprehensive estimates and standard errors that account for missing data uncertainty. Sensitivity analyses will be performed to assess the impact of different missing data assumptions, ensuring the transparency and robustness of our findings. Any subtle and mild adverse effects will be reported using a checklist.

## 3. Discussion

The present study is a double-blind, sham-controlled, parallel-group trial designed to investigate the effects of the 3-mA tDCS over the left and right DLPFC on working memory and brain physiological correlates (cortical activation, connectivity) in patients with SCZ using tDCS and fMRI. Given that cognitive deficits play a causal role in daily functioning of patients with SCZ [51] and the potential of tDCS as a cognitive boosting treatment, it is crucial to understand the mechanisms of effects of tDCS in SCZ in order to optimize the intervention. While cognitive effects of tDCS are already investigated in SCZ [8, 19], no study investigated the impact of 3 mA intensity as well as a tDCS-fMRI paradigm in SCZ.

The results of this study are helpful in optimizing tDCS protocols in SCZ and inform us of neurocognitive mechanisms underlying the interventions in several key ways. Elucidating the neurophysiological mechanisms of intensified 3-mA prefrontal tDCS will clarify how modulating frontal regions impacts local and network-wide activity to enhance cognition [23]. Understanding these neural substrates reveals how tDCS strengthens critical circuitry impaired in SCZ, as shown by frontal lobe abnormalities in this disorder [6, 7]. Identifying predictive biomarkers of tDCS response using fMRI will enable personalized targeting of tDCS to each patient’s brain activity profile. Connectivity patterns may predict behavioral and physiological responses, as dosage-dependent effects on cognition have been observed [19]. Machine learning can develop prognostic models to optimize individualized protocols. Gaining fundamental knowledge of intensified tDCS effects on SCZ pathophysiology will facilitate developing cortically-targeted cognitive treatments. Clarifying relationships between prefrontal modulation and cognitive gains provides a framework for interventions, given that frontal cognitive deficits underlie much of the disability in SCZ [3].

This study will additionally deliver initial safety and efficacy data on an intensified tDCS protocol to support larger clinical trials. Positive results could lead to rapid broader testing of a promising tool for debilitating symptoms that affect the majority of SCZ patients [1]. In summary, this research converges methodologies to optimize non-invasive brain stimulation for cognition in SCZ–a disorder where such interventions are greatly needed.

## Supporting information

S1 Checklist (DOCX)

S1 Protocol (PDF)

## List of abbreviations

SCZ: schizophrenia
tDCS: transcranial direct current stimulation
DLPFC: dorsolateral prefrontal cortex
fMRI: functional magnetic resonance imaging
BOLD: blood-oxygen-level-dependent
