# Supplementary material for: Neurocognitive effects of 3 mA prefrontal electrical stimulation in schizophrenia: A randomized sham-controlled tDCS-fMRI study protocol
Source: PLoS One. 2024 Aug 16;19(8):e0306422. doi: 10.1371/journal.pone.0306422 (PMC11329159; doi:10.1371/journal.pone.0306422)
Supplement: S1 Protocol — (PDF) [file pone.0306422.s002.pdf]

تاریخ تهیه: 75/2/2

بسمه تعالی

دانشگاه علوم پزشکی و خدمات بهداشتی درمانی ایران

Iran University of Medical Sciences

معاونت پژوهشي

Research department

پرسشنامه طرح تحقيقاتي

Checlist of research proposal

عنوان طرح: بررسی مکانیزم اثربخشی تحریک الکتریکی غیر تهاجمی مغز بر آسیب شناختی در اختلال اسکیزوفرنی: مطالعه مبتنی بر تحریک و تصویربرداری مغزی

Title: Investigating neurocognitive mechanisms of electrical brain stimulation in cognitive impairment in schizophrenia: a tDCS-fMRI study

نام و نام خانوادگي مجري طرح: دکتر رضا رستمی

Executive: Dr. Reza Rostami

دانشکده: روانشناسی و علوم تربیتی (دانشگاه تهران)

Department of Psychology, University of Tehran

1- مقدمه و معرفی طرح: Introduction

اسکیزوفرنی یکی از شدیدترین اختلالات عصب–روانپزشکی است که با پاسخ درمانی ضعیف نسبت به راهکاری درمانی موجود همراه است [1]. نقایص شناختی به ویژه نقص در حافظه کاری و کارکردهای اجرایی به میزان نسبتا بیشتری در مقایسه با علایم مثبت رایج اسکیزفرنی (توهم و هذیان) در این بیماران دیده میشود [2, 3]. این نقایص شناختی با مشکلات کارکردی و همچنین پیش آگهی منفی در این اختلال ارتباط دارند. به همین دلیل اختلال اسکیزوفرنی به عنوان یک اختلال همراه با نابهنجاری های کارکردی مغزی بویژه در ناحیه پیشانی مغز شناخته میشود که این ناحیه با نقایص شناختی در این اختلال از جمله مشکلات حافظه کاری مرتبط است [4, 5]. بر همین اساس مداخلاتی که با هدف بهبود کنترل شناختی و کارکردهای اجرایی از طریق تعدیل دادن فعالیت مغزی طراحی شده اند (با استفاده از تحریک مغزی)، اخیرا به عنوان مداخلاتی با پتانسیل درمانی در این اختلال معرف شده اند [7-9]. علایرغم نتایج نسبتا امیدوار کننده این مداخلات در اسکیزوفرنی، مکانیزمهای زیربنایی که اثربخشی آنها را تبیین میکنند هنوز شناخته نشده اند. علاوه بر این، بررسی منظم پروتوکولهای درمانی برگفته از این مداخلات هنوز جای کار دارند تا بتوانند منجر به مداخلات تاثیرگذار تر گردند.

تکنیکهای تحریک غیر تهاجمی مغز روشهای ایمنی برای تغییردادن مستقیم کارکردهای مغزی در انسان محسوب میشوند که در واقع روشی برای مطالعه علی رابطه مغز و رفتار محسوب میشوند [10]. از سویی دیگر روشهای تصویربرداری کارکردی مغزی امکان مطالعه کاکردهای مغزی را به صورت غیر مستقیم و همبستگی فراهم می آورند. ترکیب کردن این روشها ابزاری را به دست میدهد که بتوان به صورت آزمایشی و تجربی تغییراتی را در مغز (به صورت علی) ایجاد کرد و اثر آن تغییرات را در رفتار در قالب همبسته های کارکردی و فیزولوژیکی مغز بررسی کرد [10]. تحریک الکتریکی فراجمجمه ای مغز (tDCS) [11] به عنوان یک روش جدید به منظور تغییر دادن فعالیت قشری مغز که همراه با اثرات مفید شناختی در اسکیزوفرنی معرفی شده است [12] و اثرگذاری آن تا جایی که میدانیم تا حدودی به شدت و دوز تحریک بستگی دارد [2, 13].

نتایج مطالعات اخیر فیزیولوژیکی صورت گرفته بر روی نمونه های سالم با استفاد از تحریک الکتریکی فراجمجمه ای مغز نشان میدهند که تحریک آنودال (تحریکی) با شدت جریان ۳ میلی آمپر، در مقایسه با شدت تحریک ۲ و ۱ میلی آمپر، میتواند اثرات بلندمدت تر و قوی تری را بر حالت تحریک پذیری مغزی (که با تغییرات نوروپلاستیتستی همراه است) ایجاد کند [14]. از طرفی به منظور فهمیدن مکانیزم اثر تحریک الکتریکی مغز بر کارکردهای شناختی، مهم است که شبکه های کاکردی مغز را در نظر بگیریم [15]. در حالیکه مطالعات گذشته بر اهمیت شدت و دوز تحریک در اثربخشی شناختی تحریک الکتریکی فراجمجمه ای مغز در اسکیزوفرنی تاکید کرده اند، تاثیرات این پروتوکول تحریکی جدید (۳ میلی آمپر) تا کنون در اسکیزوفرنی مورد مطالعه قرار نگرفته است.

Schizophrenia is one of the most severe neuro-psychiatric disorders, which is associated with a poor therapeutic response compared to the available therapeutic strategies [1]. Cognitive deficits, especially deficits in working memory and executive functions, are seen to a greater degree of forgetfulness compared to common positive symptoms of schizophrenia (hallucinations and delusions) in these patients [2, 3]. These cognitive defects are related to functional problems as well as negative prognosis in this disorder. For this reason, schizophrenia is known as a disorder with functional brain abnormalities, especially in the frontal region of the brain, which is associated with cognitive defects in this disorder, including working memory problems [4, 5]. Accordingly, interventions designed to improve cognitive control and executive functions by modulating brain activity (using brain stimulation have recently been introduced as interventions with therapeutic potential in this disorder [7-9]. Despite the relatively promising results of these interventions in schizophrenia, the underlying mechanisms that explain their effectiveness are still unknown. In addition, regular review of treatment protocols derived from these interventions still needs to be done so that they can lead to more effective interventions.

Non-invasive brain stimulation techniques are safe methods for directly changing brain functions in humans, which are actually a method for causal study of the relationship between brain and behavior [10]. On the other hand, functional brain imaging methods provide the possibility of studying brain codes in an indirect and correlational way. Combining these methods provides a tool that can experimentally and experimentally create changes in the brain (causally) and examine the effect of thos changes on behavior in the form of functional and physiological correlates of the brain [10]. Transcranial electrical stimulation of the brain (tDCS) [11] as a new method to change the cortical

activity of the brain has been introduced with beneficial cognitive effects in schizophrenia [12] and its effect, as far as we know, is somewhat dependent on the intensity and dose. Stimulation depends.[13 ,2]

The results of recent physiological studies conducted on healthy samples using transcranial electrica stimulation of the brain show that anodal stimulation with a current intensity of 3 mA, compared t stimulation intensity of 2 and 1 mA, can have more long-term effects and create a stronger effect o brain excitability (which is associated with neuroplasticity changes) [14]. On the other hand, in order t understand the mechanism of the effect of brain electrical stimulation on cognitive functions, it i important to consider the brain's neural networks [15]. While past studies have emphasized th importance of the intensity and dose of stimulation in the cognitive effectiveness of transcrania electrical stimulation of the brain in schizophrenia, the effects of this new stimulation protocol (3 mA have not been studied in schizophrenia.

2- اهداف و فرضیات (OBJECTIVES & HYPOTHESES)

الف- اهداف کلی طرح (GENERAL OBJECTIVES):

هدف کلی این پروژه عبارتست از بررسی مکانیزم های فیزیولوژیکی اثربخشی تحریک الکتریکی ناحیه پیش پیشانی مغز با شدت ۳ میلی آمپر در بهبود حافظه کاری بیماران مبتلا به اسکیزوفرنی است.

The general goal of this project is to investigate the physiological mechanisms of the effectiveness of electrical stimulation of the prefrontal brain region with an intensity of 3 milliamps in improving the working memory of patients with schizophrenia.

ب- اهداف ویژه (SPECIFIC OBJECTIVES):

1- بررسی اثربخشی شناختی تحریک الکتریکی ناحیه پیش-پیشانی مغز با شدت ۳ میلی آمپر در عملکرد رفتاری در آزمون حافظه کاری در حین تحریک و ۲۴ ساعت بعد از تحریک الکتریکی در بیماران مبتلا به اسکیزوفرنی

2- بررسی اثرات فیزیولوژیکی تحریک الکتریکی ناحیه پیش-پیشانی مغز با شدت ۳ توسط ثبت پاسخ BOLD (تغییرات سیگنال وابسته به اکسیژن موجود در خون) قبل و بعد از تحریک در بیماران مبتلا به اسکیزوفرنی

3- بررسی میزان تغییر ارتباطات کارکردی در شبکه های مغزی بعد از تحریک الکتریکی ناحیه پیش-پیشانی مغز با شدت ۳ میلی آمپر در بیماران مبتلا به اسکیزوفرنی

1 –Investigating the cognitive effectiveness of electrical stimulation of the pre-frontal brain with an intensity of 3 milliamps in behavioral performance in the working memory test during stimulation and 24 hours after electrical stimulation in patients with schizophrenia

2 –Investigating the physiological effects of electrical stimulation of the pre-frontal brain region with intensity 3 by recording the BOLD response (signal changes dependent on oxygen in the blood) before and after the stimulation in patients with schizophrenia.

3- Investigating the amount of change in functional connections in brain networks after electrical stimulation of the pre-frontal brain region with an intensity of 3 milliamps in patients with schizophrenia

ج- فرضیات یا سئوالات پژوهش (HYPOTHESES/RESEARCH QUESTIONS):

1- **فرضیه**: تحریک الکتریکی ناحیه پیش-پیشانی مغز با شدت ۳ میلی آمپر در مقایسه با تحریک ساختگی (دارونما) باعث بهبود عملکرد بیماران مبتلا به اسکیزوفرنی در انجام آزمون حافظه کاری میشود (افزایش پاسخ صحیح)

2- **سوال ۱**: تحریک الکتریکی ناحیه پیش-پیشانی مغز با شدت ۳ میلی آمپر باعث چه تغییراتی در سیگنال وابسته به اکسیژن موجود در خون در گروه ازمایشی در مقایسه با گروه دارونما میشود؟

3 - **سوال ۲**: تحریک الکتریکی ناحیه پیش-پیشانی مغز با شدت ۳ میلی آمپر باعث چه تغییراتی در ارتباطات کارکردی در گروه ازمایشی در مقایسه با گروه دارونما میشود میشود؟

-1Hypothesis: electrical stimulation of the pre-frontal area of the brain with an intensity of 3 milliamps compared to sham stimulation (placebo) improves the performance of patients with schizophrenia in working memory tests (increasing correct answers)
-2Question 1: Electrical stimulation of the pre-frontal area of the brain with an intensity of 3 milliamps causes changes in the oxygen-dependent signal in the blood in the experimental group compared to the placebo group?
-3Question 2: Electrical stimulation of the pre-frontal area of the brain with an intensity of 3 milliamps causes what changes in functional communication in the experimental group compared to the placebo group?

3- روش ها و مواد(MATERIALS AND METHODS) :

د- جامعه و نمونه پژوهش:

جامعه: بیماران اسکیزوفرنی غیر کاتاتونیک دارای تخریب شناختی در شهر تهران

نمونه: بیماران اسکیزوفرن در بیمارستان رازی تهران و کلینیک های تخصصی اسکیزوفرنی

A- research sample:

Sample: non-catatonic schizophrenic patients with cognitive impairment in Tehran
Example: Schizophrenic patients in Tehran's Razi Hospital and specialized schizophrenia clinics

ه - روش نمونه گیری و حجم نمونه:

روش نمونه گیری به صورت در دسترس است. اندازه نمونه 30 نفر میباشد که در دوگروه 15 نفره آزمایشی و کنترل تقسیم میشوند. حجم نمونه بر اساس مطالعات مشابه صورت گرفته بر روی اختلال اسکیزوفرنی با مداخله مشابه (گوتسن و همکاران، 2021) و همچنین محاسبه حجم نمونه با استفاده از روش تحلیل قدرت (Power analysis) نیز محاسبه گردیده است که حداقل نمونه برای این طرح دارای گروه موازی با اندازه اثر متوسط (f=0.3) 30 نفر میباشد.

B- Sampling method and sample size:

30 Patients will be recruited and will be randomly assigned to active and sham groups. The inclusion criteria are (1) men or women (18-50 yrs old) with a DSM 5 SCZ diagnosis, (2) significant cognitive impairment (1 SD below the norm on the working memory/executive functioning task performance), (3) feasibility for tDCS interventions according to safety guidelines, (4) stable medication regime especially the classical neuroleptics and all CNS-activating medications, if taken, 4-6 weeks before the experiment, (5) right-handed, and (6) ability to provide informed consent. The exclusion criteria include pregnancy, alcohol or substance dependence, history of seizure, neurological disorder, or head injury. The required sample size for this randomized, parallel-group design (f=0.35 equivalent to $\eta p^2=0.10$, $\alpha=0.05$, power=0.95) would be 24 according to the results of a power analysis. We propose to include an additional 6 to account for a 20% potential dropouts during the study. The smoking rate will be documented and controlled, as most SCZ patients are smokers and exclusion of smokers is not feasible.
.

و- روش گردآوری اطلاعات و مشخصات ابزار گردآوری اطلاعات:

اطلاعات رفتاری: از آزمون رفتاری حافظه کاری n-back به منظور ثبت اطلاعات رفتاری (شامل زمان واکنش و میزان پاسخ صحیح) استفاده میشود

اطلاعات فیزیولوژیکی مغز: اطلاعات فیزیولوژیکی بوسیله ثبت پاسخ BOLD و ارتباطات کارکردی توسط اندازه گیری fMRI در حالت استراحت قبل و بعد از مداخله گرداوری میشوند.

C- data collection and meassures:

Behavioral data: The n-back working memory behavioral test is used to record behavioral information (including reaction time and correct response rate).

Physiological data: Physiological information is collected by recording the BOLD response and functional communication by measuring fMRI in the resting state before and after the intervention.

ز- نحوه انجام کار:

بعد از گزینش آزمودنی ها با توجه به ملاکهای ورود و خروج و اخذ رضایت نامه کتبی، آزمودنی ها به صورت تصادفی به دوگروه آزمایشی و کنترل تقسیم میشوند. هر آزمودنی قبل از شروع آزمایش آزمون حافظه کاری را قبل از مداخله انجام میدهد (خط پایه). یک هفته بعد آزمودنی ها توسط آزمایشگر و افراد مربوطه در آزمایشگاه ملی مغز حاضر شده و بسته به گروه مداخله ۲۰ دقیقه تحریک الکتریکی واقعی یا ساختگی در حالی که در دستگاه fMRI قرار دارند دریافت میکنند در حالیکه تکلیف آزمون حافظه کاری را در حین تحریک انجام میدهند. قبل از انجام یک مداخله اسکن ساختاری مغز (با وزن T1) و یک ثبت در حالت استراحت fMRI صورت میگرد و پس از انجام تحریک مغزی مجددا یک ثبت دیگر fMRI در حالت استراحت صورت میگیرد. آزمودنی ها سپس مرخص شده و به سرپرست مربوطه جهت مراجعت به منزل یا بیمارستان تحویل داده میوشند. ۲۴ ساعت بعد از انجام تحریک آزمودنی ها مجددا تکلیف رفتاری حافظه کاری را در محل سکونت خود و از طریق لپ تاپ انجام میدهند. مراجعه و بازگشت آزمودنی ها توسط وسیله نقلیه فراهم شده توسط آزمایشگران صورت میگیرد. همچنین مطالعه به صورت دو سرکور است بدین شکل که آزمودنی ها از نوع تحریک دریافتی (ساختگی یا دارونما) اطلاع ندارند. تکنیسین هایی که مداخله و ثبت را انجام میدهند نیز از فرضیه ها و سوالات پژوهشی اطلاعی ندارند. داده ها توسط آزمایشگری مستقل تحلیل میشود. هر آزمودنی بابت حضور در مطالعه مبلغی را دریافت میکند (نمودار 1).

D- Procedure: After selecting the subjects according to the entry and exit criteria and obtaining written consent, the subjects are randomly divided into experimental and control groups. Before starting the experiment, each subject performs a working memory test before the intervention (baseline). One week later, the subjects were brought to the National Brain Laboratory by the experimenter and relevant people, and depending on the intervention group, they received 20 minutes of real or fake electrical stimulation while they were in the fMRI machine, while they performed the task of the working memory test during the stimulation. . Before the intervention, a structural brain scan (with T1 weighting) and an fMRI recording in the resting state are performed, and after the brain stimulation, another fMRI recording is performed in the resting state. The subjects are then discharged and handed over to the relevant supervisor to go home or to the hospital. 24 hours after the stimulation, the subjects will perform the behavioral task of working memory again at their place of residence and through a laptop. Visiting and returning of the subjects is done by the vehicle provided by the experimenters. Also, the study is double-blind so that the subjects do not know the type of stimulation received (fake or placebo). The technicians who perform the intervention and registration are also not aware of the hypotheses and research questions. The data is analyzed by an independent experimenter. Each subject receives an amount for participating in the study (Fig. 1).

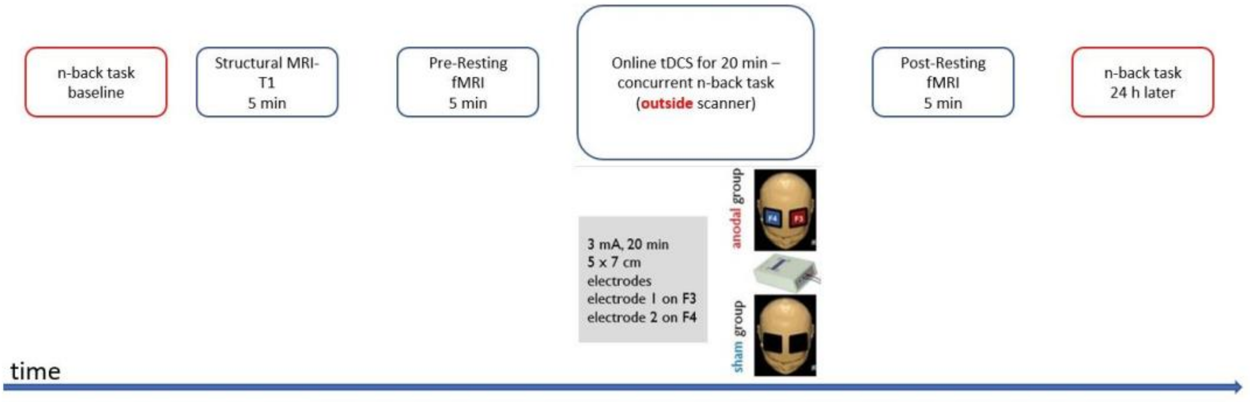

Fig. 1: The course of study

ح- روش تجزیه و تحلیل اطلاعات:

تجزیه و تحلیل اطلاعات رفتاری توسط نرم افزار SPSS و R صورت میگیرد. اطلاعات تصویربردرای مغزی توسط پکیج های رایج تحلیل داده های تصویربرداری از جمله SPM و یا FSL صورت میگیرد. روش آماری مورد استفاده برای تحلیل آماری داده ای مذکور با توجه به اینکه طرح آزمایشی با گرووهای موازی است، آزمون تحلیل واریانس با اندازه گیری مکرر برای طرحهای بین گروهی خواهد بود (Mixed model repeated meassures ANOVA)

E- Statistical analysis:

Behavioral data analysis is done by SPSS and R software. Brain imaging data is processed by common imaging data analysis packages such as SPM or FSL. The statistical method used for the statistical analysis of the mentioned data, considering that the experimental design is with parallel grooves, will be the variance analysis test with repeated measures for between-group designs (Mixed model repeated measures ANOVA).

1- Participants: 30 Patients will be recruited and will be randomly assigned to active and sham groups. The inclusion criteria are (1) men or women (18-50 yrs old) with a DSM 5 SCZ diagnosis, (2) significant cognitive impairment (1 SD below the norm on the working memory/executive functioning task performance), (3) feasibility for tDCS interventions according to safety guidelines, (4) stable medication regime especially the classical neuroleptics and all CNS-activating medications, if taken, 4-6 weeks before the experiment, (5) right-handed, and (6) ability to provide informed consent. The exclusion criteria include pregnancy, alcohol or substance dependence, history of seizure, neurological disorder, or head injury. The required sample size for this randomized, parallel-group design (f=0.35 equivalent to $\eta p^2=0.10$, $\alpha=0.05$, power=0.95) would be 24 according to the results of a power analysis. We propose to include an additional 6 to account for a 20% potential dropouts during the study. The smoking rate will be documented and controlled, as most SCZ patients are smokers and exclusion of smokers is not feasible.

2- Intervention: A single session of prefrontal tDCS will be conducted. The tDCS montage in both, active and sham conditions will be anodal left DLPFC (F3)- cathodal right DLPFC (F4) (3 mA, 20 min). The protocol is based on recent findings from the motor cortex model in healthy individuals showing longer-lasting and stronger aftereffects compared to conventional protocols[17]. To achieve efficient blinding, topical anesthetic cream will be applied under the electrode areas in line with previous studies[18].

3- Behavioral measure: A computerized version of the n-letter back working memory task, will be employed during the experiment. The working memory paradigm includes 1 and 3-back conditions to account for working memory load. The order of stimuli presentation will be randomized. The task takes 6 minutes for each block (12 min in total) to complete.

4- Functional MRI acquisition and analyses: The fMRI data will be acquired with a 3T PRISMA Scanner (Siemens, Germany) and include a T1-weighted sequence (5min), a pre-intervention resting-state fMRI (5 min) followed by post-intervention fMRI sequences (20 min). Functional MRI data will be analyzed with a robust processing pipeline with SPM and FSL[19]. Right and left DLPFC will be used to construct brain functional connectivity patterns. The changes in these patterns during treatment conditions will be compared.

5- Procedure: After recruiting patients, receiving written informed consent, at screening participants' chronotypes[20], participants will undergo baseline task performance before the intervention. One week later, they attend the National Brain Mapping Laboratory (NBML) at the University of Tehran for the pre-intervention resting-state fMRI acquisitions. They will undergo the tDCS intervention (active or sham) during which they perform the working memory task (online stimulation). Right after the stimulation participants go to the scanner again for the post-intervention resting fMRI. They will perform the task 24 h later again

4- سابقه علمی پژوهشهای انجام شده و فهرست منابع (LITERATURE REVIEW & REFERENCES):

1. Jääskeläinen, E., et al., *A Systematic Review and Meta-Analysis of Recovery in Schizophrenia.* ***Schizophrenia Bulletin***, 2012. **39**(6): p. 1296-1306.
2. Orlov, N.D., et al., *Stimulating thought: a functional MRI study of transcranial direct current stimulation in schizophrenia.* ***Brain***, 2017. **140**(9): p. 2490-2497.
3. Kerns, J.G., et al., *Executive Functioning Component Mechanisms and Schizophrenia.* ***Biological Psychiatry***, 2008. **64**(1): p. 26-33.
4. Minzenberg, M.J., et al., *Meta-analysis of 41 Functional Neuroimaging Studies of Executive Function in Schizophrenia.* ***Archives of General Psychiatry,*** 2009. **66**(8): p. 811-822.
5. Marenco, S., et al., *Investigation of Anatomical Thalamo-Cortical Connectivity and fMRI Activation in Schizophrenia.* ***Neuropsychopharmacology***, 2012. **37**(2): p. 499-507.
6. Aleman, A., et al., *Moderate effects of noninvasive brain stimulation of the frontal cortex for improving negative symptoms in schizophrenia: Meta-analysis of controlled trials.* ***Neuroscience & Biobehavioral Reviews***, 2018. **89**: p. 111-118.
7. Salehinejad, M.A., et al., *Cognitive control dysfunction in emotion dysregulation and psychopathology of major depression (MD): Evidence from transcranial brain stimulation of the dorsolateral prefrontal cortex (DLPFC).* ***Journal of Affective Disorders***, 2017. **210**: p. 241-248.
8. Alizadehgoradel, J., et al., *Repeated stimulation of the dorsolateral-prefrontal cortex improves executive dysfunctions and craving in drug addiction: A randomized, double-blind, parallel-group study.* **Brain Stimulation**, 2020. **13**(3): p. 582-593.
9. Molavi, P., et al., *Repeated transcranial direct current stimulation of dorsolateral-prefrontal cortex improves executive functions, cognitive reappraisal emotion regulation, and control over emotional processing in borderline personality disorder: A randomized, sham-control, parallel-group study.* ***Journal of Affective Disorders***, 2020..
10. Polanía, R., M.A. Nitsche, and C.C. Ruff, *Studying and modifying brain function with non-invasive brain stimulation.* ***Nature Neuroscience***, 2018. **21**(2): p. 174-187.
11. Nitsche, M. and W. Paulus, *Excitability changes induced in the human motor cortex by weak transcranial direct current stimulation.* ***The Journal of Physiolog***y, 2000. **527**(3): p. 633-639.
12. Guttesen, L.L., et al., *Repetitive transcranial magnetic stimulation and transcranial direct current stimulation for auditory hallucinations in schizophrenia: Systematic review and meta-analysis.* ***Journal of Psychiatric Research***, 2021. **143**: p. 163-175.
13. Hoy, K.E., et al., *An investigation into the effects of tDCS dose on cognitive performance over time in patients with schizophrenia.* ***Schizophrenia Research***, 2014. **155**(1): p. 96-100.
14. Agboada, D., et al., *Expanding the parameter space of anodal transcranial direct current stimulation of the primary motor cortex.* ***Scientific Reports***, 2019. **9**(1): p. 18185.
15. Peña-Gómez, C., et al., *Modulation of large-scale brain networks by transcranial direct current stimulation evidenced by resting-state functional MRI.* ***Brain Stimulation***, 2012. **5**(3): p. 252-263.
16. Agboada, D., et al., *Induction of long-term potentiation-like plasticity in the primary motor cortex with repeated anodal transcranial direct current stimulation – Better effects with intensified protocols?* ***Brain Stimulation***, 2020. **13**(4): p. 987-997.
17. Guleyupoglu, B., et al., *Reduced discomfort during high-definition transcutaneous stimulation using 6% benzocaine.* ***Frontiers in Neuroengineering***, 2014. **7**(28).
18. Parkes, L., et al., *An evaluation of the efficacy, reliability, and sensitivity of motion correction strategies for resting-state functional MRI.* ***NeuroImage***, 2018. **171**: p. 415-436.

صحت مطالب مندرج در پرسشنامه را تایید می‌نمایم.

امضای مجری طرح: رضا رستمی

.We confirm the accuracy of the contents in the questionnaire

Signature of project manager: Reza Rostami
